# The association between the degree of frailty and the risk of hospital related adverse events in older acutely admitted patients

**DOI:** 10.1093/ageing/afag175

**Published:** 2026-06-18

**Authors:** Faris Alotaibi, Bradly Manktelow, Abdullah Alshibani, Adam Linton, Jay Banerjee

**Affiliations:** Imam Abdulrahman Bin Faisal University, College of Applied Medical Sciences, Emergency Medical Care Department. Dammam, Saudi Arabia; University of Leicester, College of Life Sciences, School of Healthcare, Leicester, England, United Kingdom of Great Britain and Northern Ireland; University of Leicester, College of Life Sciences, School of Healthcare, Leicester, England, United Kingdom of Great Britain and Northern Ireland; King Saud bin Abdulaziz University for Health Sciences, College of Applied Medical Sciences, Emergency Medical Services Department, Riyadh, Saudi Arabia; University of Leicester, College of Life Sciences, Health Sciences, Leicester, United Kingdom of Great Britain and Northern Ireland; King Abdullah International Medical Research Center, Riyadh, Saudi Arabia; University Hospitals of Leicester NHS Trust, Department of Emergency and Specialist Medicine (ESM), Leicester, England, United Kingdom of Great Britain and Northern Ireland; University Hospitals of Leicester NHS Trust, Department of Emergency and Specialist Medicine (ESM), Leicester, England, United Kingdom of Great Britain and Northern Ireland; University of Leicester, College of Life Sciences, Global Health, Lifestyle and Metabolic Health, Leicester, United Kingdom of Great Britain and Northern Ireland

**Keywords:** frailty, patient safety, adverse events, Datix, older people

## Abstract

**Introduction:**

Frailty is an age-related syndrome characterised by increased vulnerability. Hospital-related adverse events (AEs) are complications not directly caused by patient’s conditions. While age has been widely studied, less is known about the effect of the degree of frailty on in-hospital AEs among older adults.

**Methods:**

This single-centre observational study used routinely collected data from a university teaching hospital in the UK October 2017 and 2023. Patients aged ≥65 admitted via the Emergency d.

Department (ED) with a recorded frailty score at ED using the Clinical Frailty Scale (CFS) were included. AE types were in-hospital falls, pressure ulcers, moisture damage, infections, medication-related events, self-harm, discharge/transfer incidents and hospital injuries. Poisson regression models were used to estimate associations between frailty and AEs, adjusting for age, sex, ethnicity, ED wait time and Inpatient (IP) length of stay.

**Results:**

Out of 369 798 admissions, 158 470 were included, and the degree of frailty was significantly associated with increased risk of AE. Compared to non-frail patients, adjusted RRs for those with CFS 7–9: in-hospital falls (RR = 1.46), pressure ulcers (RR = 2.75), moisture damage (RR = 3.56), infections (RR = 2.03), discharge/transfer incidents (RR = 2.42) and hospital injuries (RR = 1.66). While AE risk increased with frailty, some AE showed a slight decline in the severely frail group.

**Conclusion:**

The degree of frailty was associated with the risk of hospital-related AEs in older adults. Higher degree of frailty was associated with multiple AE types, supporting early frailty identification, targeted preventive strategies and tailored care for risk reduction.

## Key Points

- This study analysed more than 158 000 admissions records of acutely admitted older patients.
- The degree of frailty was significant and independent predictor of the risk of hospital related adverse events.
- A limitation of this study is that it was at one centre which limit generalisability.

## Introduction

The global population of older adults is increasing rapidly. The United Nations (UN) reported 703 million people aged 65 and above in 2019 and anticipates this number could reach 1 billion by 2050 [1]. People are living longer, and average life expectancy has increased due to advances in public health, improvements in sanitation and clean water, better nutrition and the development of vaccines and antibiotics [2–4]. The increase in expected life span is associated with rising healthcare demands. In the UK, people aged 65 years and older accounted for 62% of hospital bed occupancy in 2014/15 [5]. Frailty is one of the most common age-related syndromes, affecting approximately one in ten older adults, with a higher prevalence observed among women [6].

Frailty is a clinical syndrome characterised by reduced physiological reserve and increased vulnerability to stressors [7]. It is associated with adverse outcomes, including functional decline, complications and mortality [8]. Various tools are used to assess frailty, including Fried’s phenotype model [7], the Frailty Index based on cumulative deficits [9] and the Clinical Frailty Scale (CFS). CFS is a widely used clinician-rated tool that captures functional status [10]. CFS is particularly attractive for use by non-aged-care specialists because of its simplicity, speed and practicality in busy clinical settings like the ED, with evidence suggesting good predictive value [11, 12].

While age has been widely studied in predicting the risk of healthcare-related adverse events (AE), less is known regarding the association between frailty and the risk of AEs among acutely admitted patients undergoing unplanned hospitalisation requiring urgent medical care [13]. An AE is any unintended complication or injury that occurs during healthcare provision that is unrelated to the patient’s underlying medical condition [14]. AEs are associated with prolonged hospital stay, disability, or even death [15]. It has been estimated that AEs occur in 1 in every 10 patients [16], and 50% of AEs are possibly preventable [17]. There are many methods to report and identify AEs in hospitalised patients, including voluntary incident reporting, chart review, or patient interviews [18]. Datix, a web-based incident reporting system, is commonly used in NHS hospitals to report, investigate and resolve AE incidents [19]. A growing body of evidence has demonstrated an association between frailty, measured using various frailty assessment tools, and the incidence of AEs and poor clinical outcomes among older adults across different healthcare settings, including surgical, medical and intensive care environments [20]. However, there remains a lack of evidence examining the association between frailty and hospital-related AEs in older acutely admitted patients. Therefore, this study aims to investigate the association between different frailty degrees, as measured by the CFS, and the risk of specific types of hospital-related AEs in older acutely admitted patients. This understanding can support clinical decision-making, accurate risk assessment and in tailoring care to prevent potential harm.

## Method

### Setting

This study was conducted in a large university teaching hospital in the East Midlands of the United Kingdom, with approximately 240 000 annual patient attendances, including 50 000 older people. The acute trust covers a catchment area of 1.1 million people and has a single-site ED [21]. In October 2017, the hospital began recording frailty status of older people aged 65 years and older using the CFS in the electronic health record. The hospital uses the Datix incident reporting system for local reporting and learning from AEs. Datix, is a web-based incident reporting system commonly used in the NHS to report, investigate and resolve AE incidents [19].

### Study type and sample

A retrospective observational study was conducted among patients aged 65 years and older who acutely presented to the ED or were admitted through the ED to inpatient (IP) care. Frailty, assessed in the ED using the CFS, was arbitrarily categorised into non-frail (CFS 1–3), and frail which included subcategorises of mild (CFS 4–5), moderate (CFS 6) and severe (CFS 7–9) frailty to improve statistical stability and facilitate clinical interpretation. Admissions with a reported frailty score of patients aged 65 and older were included. AEs associated with these admissions were extracted from the Datix records. AE were grouped into ten types: medication errors, adverse drug reactions, hospital-acquired infections, in-hospital falls, hospital injuries, pressure ulcers, moisture-associated skin damage, patient abuse, self-harm and incidents related to discharge or transfer (see Appendix 1). To assess potential selection bias related to missing frailty data, a comparative analysis was performed between admissions with and without a recorded CFS among patients aged ≥65 years presenting to the ED. Patient characteristics compared included age, sex, ethnicity, ED waiting time, IP admission status, length of stay, discharge destination and in-hospital mortality.

### Statistical analysis

Patient characteristics for this analysis were summarised across the four frailty categories defined previously. Total admission in each frailty category was reported as percentages of the total admissions included. Patient demographics (age, sex and ethnicity), ED measures (wait time in minutes and ED admission to IP indicator) and IP measures (length of stay in nights, and discharge destination) was summarised by frailty categories. Details of the grouped discharge destinations are listed in Appendix 2. In-hospital mortality rates were also compared across the frailty groups. Continuous variables were summarised using the mean and standard deviation (SD) or the median and interquartile range (IQR), depending on normality of distribution. Categorical variables were presented as counts and as percentages of the total number of admissions in each frailty category.

The association between the degree of frailty and the risk of AE by type was analysed using Poisson regression models to estimate the relative risks (RR) with 95% confidence intervals (CIs). First, a crude model was fitted with frailty categories as the sole predictor to assess the unadjusted association between the degree of frailty and AE risk. The number of patients having at least one of each AE type and the rate per 1000 admissions was calculated for all AE types by degree of frailty using the number of admissions within each frailty category as the denominator. The relative risk was calculated by comparing the rate for each frailty category to the non-frail group. Next, adjusted models were developed by using frailty categories as a predictor and accounting for potential confounders: age, sex, ethnicity, ED wait time and IP length of stay. Ethnicity was included as a potential confounder due to previously reported differences in frailty prevalence and patient safety outcomes across ethnic groups [22, 23]. Adjusted relative risks were calculated and a significance level of *P* < .05 used to indicate statistical significance. As a sensitivity analysis, the Poisson regression models were re-estimated using robust standard errors clustered at the patient level to account for multiple admissions from the same individual.

## Results

A total of 158 470 acute admissions with a recorded frailty score were included out of total 369 798 total acute admissions of older people to the ED, over the period of October 2017 to October 2023 (Table 1). Of the total admissions, 88,369 records missing ED records were excluded as frailty was not assessed at ED presentation and therefore could not be determined for these admissions. Among the remaining admissions with ED records, 158 470 (56.3%) had a recorded CFS and 122 959 (43.7%) had no frailty assessment. Admissions with recorded frailty were older (median 80 vs 76 years), had longer ED waiting times, higher admission rates, longer IP stays and higher in-hospital mortality. Differences in ethnic distribution were modest, and sex distribution was similar between groups.

**Table 1:** Patient characteristics by frailty degree.

Patient characteristics	Non-frail CFS 1–3	Mild frailty CFS 4–5	Moderate frailty CFS 6	Severe frailty CFS 7–9
Total Admissions N (%)	35 691 (22.5%)	62 581 (39.5%)	37 068 (23.4%)	23 130 (14.6%)
Median Age in years (IQR)	74 (69–80)	80 (74–86)	84 (78–89)	84 (77–90)
Female % (N)	48.9% (*n* = 17 455)	53.3% (*n* = 33 333)	58.3% (*n* = 21 599)	58.4% (*n* = 13 519)
White Ethnicity % (N)	82.7% (*n* = 29 536)	83.4% (*n* = 52 177)	85.1% (*n* = 31 551)	83.8% (*n* = 19 382)
Median ED Wait Time in Minutes (IQR)	334 (202–556)	475 (293–771)	516 (326–815)	460 (292–730)
Median IP Length of Stay in Nights (IQR)	3 (1–7)	5 (2–11)	7 (3–14)	7 (3–14)
Mortality during admission % (N)	1.5% (*n* = 553)	4.4% (*n* = 2745)	7.6% (*n* = 2827)	11.9% (*n* = 2750)
Admitted from ED to IP % (N)	54.3% (*n* = 19 387)	74.1% (*n* = 46 377)	79.5% (*n* = 29 478)	76.8% (*n* = 17 758)
Admissions with at least one AEs % (N)	3.0% (*n* = 1072)	7.8% (*n* = 4870)	11.5% (*n* = 4257)	10.7% (*n* = 2466)
Discharge destination % (N)				
Home	94.16% (*n* = 16 858)	84.54% (*n* = 35 170)	70.22% (*n* = 17 892)	64.2% (*n* = 9192)
Hospital transfer/rehabilitation	4.35% (*n* = 779)	10.0% (*n* = 4144)	14.3% (*n* = 3634)	9.5% (*n* = 1361)
Care home/residential placement	1.31% (*n* = 235)	5.1% (*n* = 2117)	15.1% (*n* = 3854)	25.7% (*n* = 3677)
Hospice	0.11% (*n* = 20)	0.20% (*n* = 82)	0.18% (*n* = 46)	0.41% (*n* = 59)
Secure psychiatric or custodial placement	0.07% (*n* = 12)	0.21% (*n* = 87)	0.21% (*n* = 53)	0.24% (*n* = 34)

The degree of frailty increased progressively with age, from a median of 74 years (IQR 69–80) in non-frail patients to 84 years (IQR 77–90) in severely frail. The proportion of females rose from 48.91% in non-frail to 58.45% in severely frail. White ethnicity outweighed other ethnicities with 82.75 in non-frail and peaking to 85.1 in moderately frail, followed by Asian ethnicity peaking at 13.04% in mildly frail patients, while other ethnicities like Black, Mixed and Other un-specified ethnicities accounted for less than 2% each.

ED wait time increased with the degree of frailty, from a median of 334 minutes (IQR 202–556) in non-frail patients to 516 minutes (IQR 326–815) in moderate frailty, then declined slightly in the severe group to 460 minutes (IQR 292–730). Admission from ED to IP wards followed a similar pattern and increased from 54.3% in non-frail to 79.5% in moderately frail then declined in severely frail patients.

The length of IP stay increased consistently with the degree of frailty rising from 3 (IQR 1–7) in non-frail to 7 (IQR 3–14) nights in severely frail. In-hospital mortality followed a similar pattern; it rose markedly with the degree of frailty from 1.5% in non-frail patients to 11.9% in the severe frailty group. Discharge destination differed by frailty group. Return home became less common as frailty increased, falling from 94.2% in CFS 1–3 to 64.2% in CFS 7–9. By contrast, discharge to a care home or residential setting became progressively more frequent with increasing frailty. Discharge to secure psychiatric or custodial settings was rare but occurred slightly more often in the frailer groups. Hospice discharges were uncommon across all frailty groups (<0.5%).

Admissions with at least one AE increased with the degree of frailty, it increased from 3.0% (*n* = 1072) in non-frail patients to 7.8% (*n* = 4870) in mildly frail and reached 11.5% (*n* = 4257) in moderately frail patient, then slightly declined to 10.7% (*n* = 2466) in severely frail patients.

### The association between the degree of frailty and AE types

**Figure 1:**
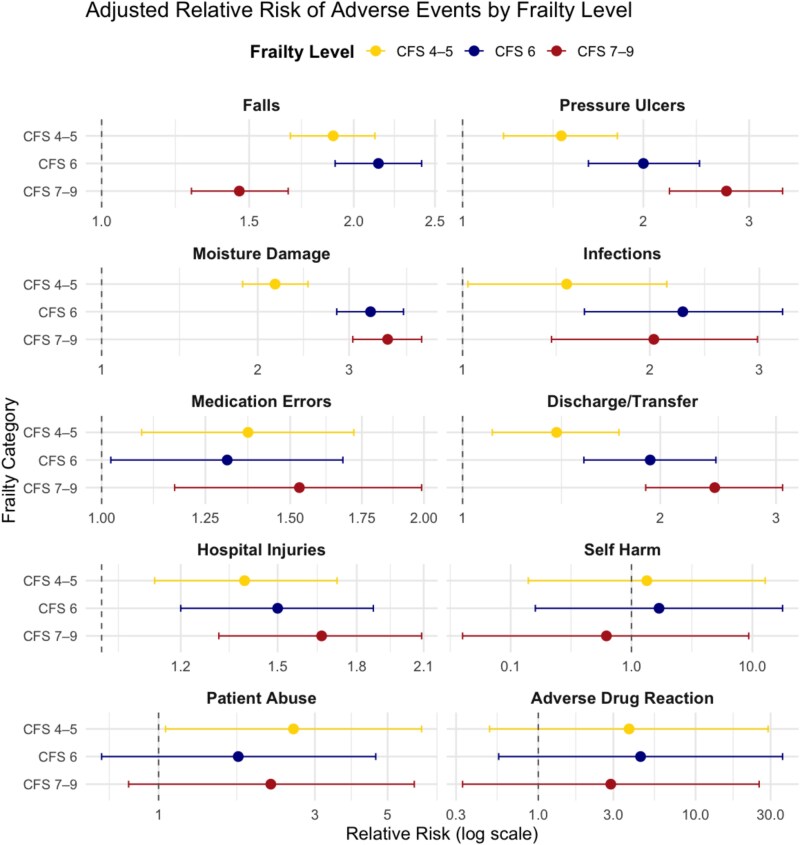
Adjusted RR of specific AEs by frailty category.

Pressure ulcers, moisture-associated skin damage, in-hospital injuries and discharge/transfer incidents increased with the degree of frailty. Pressure ulcer adjusted rates were nearly three times higher in severely frail than non-frail patients (adjusted RR = 2.75, *P* < .001), with adjusted risks rising across all frailty categories. Moisture-associated skin damage showed the same graded pattern (severely frail vs non-frail: adjusted RR = 3.56, *P* < .001), as did in-hospital injuries (adjusted RR 1.66, *P* < .001). Discharge/transfer incidents also rose with frailty, with an adjusted RR of 2.42 in severe frailty compared to non-frail (*P* < .001) (Table 2; Figure 1).

**Table 2:** Risk of AEs per frailty. Adjusted for age, ethnicity, sex, ED wait time (minutes) and IP length of stay (nights).

Frailty score by CFS	N of admission with at least one AE	Crude rate per 1000 admissions (95% CI)^a^	Crude RR (95% CI)	Adjusted RR (95% CI)^b^	Adjusted *P*-value
In-hospital Falls					
CFS 1–3	422	11.8 (10.8–13.0)	ref	ref	
CFS 4–5	2032	32.5 (31.1–33.9)	2.75 (2.47–3.05)	1.89 (1.68–2.12)	<.001
CFS 6	1586	42.8 (40.8–44.9)	3.62 (3.25–4.02)	2.14 (1.90–2.41)	<.001
CFS 7–9	636	27.5 (25.5–29.7)	2.33 (2.06–2.63)	1.46 (1.28–1.67)	<.001
Pressure Ulcers					
CFS 1–3	130	3.6 (3.1–4.3)	ref	ref	
CFS 4–5	559	8.9 (8.2–9.7)	2.45 (2.03–2.97)	1.46 (1.17–1.81)	<.001
CFS 6	546	14.7 (13.6–16.0)	4.04 (3.34–4.89)	2.00 (1.62–2.48)	<.001
CFS 7–9	421	18.2 (16.6–20)	5.00 (4.11–6.08)	2.75 (2.21–3.41)	<.001
Moisture Associated Skin Damage					
CFS 1–3	226	6.3 (5.6–7.2)	ref	ref	
CFS 4–5	1326	21.2 (20.1–22.3)	3.35 (2.91–3.85)	2.16 (1.87–2.5)	<.001
CFS 6	1398	37.7 (35.8–39.7)	5.96 (5.18–6.85)	3.30 (2.84–3.82)	<.001
CFS 7–9	868	37.5 (35.2–40.1)	5.93 (5.12–6.85)	3.56 (3.05–4.14)	<.001
Adverse Drug Reaction					
CFS 1–3	1	0 (0–0.2)	ref	ref	
CFS 4–5	11	0.2 (0.1–0.3)	6.27 (0.81–48.59)	3.78 (0.49–28.91)	.21
CFS 6	8	0.2 (0.1–0.4)	7.70 (0.96–61.58)	4.47 (0.56–35.76)	.17
CFS 7–9	3	0.1 (0–0.4)	4.63 (0.48–44.5)	2.89 (0.33–25.37)	.37
Medication Errors					
CFS 1–3	111	3.1 (2.6–3.7)	ref	ref	
CFS 4–5	347	5.5 (5–6.2)	1.78 (1.44–2.21)	1.37 (1.09–1.72)	.006
CFS 6	219	5.9 (5.2–6.7)	1.90 (1.51–2.39)	1.31 (1.02–1.68)	.03
CFS 7–9	153	6.6 (5.6–7.7)	2.13 (1.67–2.71)	1.53 (1.17–1.99)	.001
Hospital-Acquired Infections					
CFS 1–3	42	1.2 (0.9–1.6)	ref	ref	
CFS 4–5	199	3.2 (2.8–3.7)	2.70 (1.94–3.77)	1.47 (1.02–2.13)	.03
CFS 6	219	5.9 (5.2–6.7)	5.02 (3.61–6.98)	2.26 (1.57–3.27)	<.001
CFS 7–9	105	4.5 (3.8–5.5)	3.86 (2.7–5.51)	2.03 (1.39–2.98)	<.001
Patient Abuse					
CFS 1–3	7	0.2 (0.1–0.4)	ref	ref	
CFS 4–5	44	0.7 (0.5–0.9)	3.58 (1.61–7.96)	2.58 (1.05–6.35)	.032
CFS 6	21	0.6 (0.4–0.9)	2.89 (1.23–6.79)	1.75 (0.67–4.6)	.24
CFS 7–9	15	0.6 (0.4–1.1)	3.31 (1.35–8.11)	2.20 (0.81–6.03)	.11
Self-Harm					
CFS 1–3	1	0.0 (0.0–0.2)	ref	ref	
CFS 4–5	5	0.1 (0.0–0.2)	2.85 (0.33–24.41)	1.34 (0.14–12.78)	.79
CFS 6	5	0.1 (0.1–0.3)	4.81 (0.56–41.2)	1.69 (0.16–17.8)	.64
CFS 7–9	1	0.0 (0.0–0.2)	1.54 (0.1–24.67)	0.62 (0.04–9.34)	.74
Discharge & Transfer					
CFS 1–3	114	3.2 (2.7–3.8)	ref	ref	
CFS 4–5	380	6.1 (5.5–6.7)	1.90 (1.54–2.34)	1.39 (1.11–1.73)	.004
CFS 6	349	9.4 (8.5–10.5)	2.95 (2.39–3.64)	1.93 (1.53–2.43)	<.001
CFS 7–9	261	11.3 (10–12.7)	3.53 (2.84–4.4)	2.42 (1.9–3.07)	<.001
In-Hospital Injuries					
CFS 1–3	120	3.4 (2.8–4.0)	ref	ref	
CFS 4–5	478	7.6 (7.0–8.4)	2.27 (1.86–2.77)	1.39 (1.13–1.72)	.002
CFS 6	368	9.9 (9.0–11.0)	2.95 (2.4–3.63)	1.50 (1.2–1.87)	<.001
CFS 7–9	233	10.1 (8.9–11.4)	3.00 (2.41–3.73)	1.66 (1.31–2.09)	<.001

^a^The rate per 1000 was calculated out of total admissions in each frailty category.

^b^Adjusted for age, sex, ethnicity, ED wait time and IP length of stay.

In-hospital falls reached their highest incidence among moderately frail patients, where the adjusted rate was twice that of the non-frail group (adjusted RR = 2.14, *P* < .001). Hospital-acquired infections were elevated in all frailty categories compared to non-frail patients (moderately frail vs non-frail: adjusted RR = 2.26, *P* < .001). Medications errors also had higher rates across all frailty categories compared to the non-frail patients (severely frail vs non-frail: adjusted RR = 1.53, *P* = .001).

Adverse drug reactions and patient abuse showed increased risk among frail patients, but this did not reach statistical significance. (Table 2; Figure 1). There was no evidence of differences in risk of self-harm, but the number of admissions where self-harm occurred was very small. (Table 2; Figure 1). Sensitivity analyses using patient-level clustered robust standard errors showed similar effect estimates and confidence intervals, suggesting that multiple admissions from the same patient did not materially affect the findings.

## Discussion

This study found frailty to be a strong and an independent predictor of AEs among acutely admitted older adults, even after adjusting for age, sex, ethnicity, ED wait time and IP stay. Frailty increased progressively with age, likely reflecting physiological decline and reduced reserves that heighten vulnerability to stressors [24]. The rising proportion of females with frailty is multifactorial [25]. While White patients formed the largest group across all frailty categories, this appears to reflect the sample’s demographic structure rather than meaningful differences in frailty distribution. Examination of frailty categories within each ethnic group showed broadly similar proportions across groups. Higher frailty degree was also associated with a greater likelihood of IP admission, longer ED wait times, extended hospital stays and increased mortality, which is consistent with prior research showing worse outcomes and higher mortality in frail patients [26, 27]. Discharge to non-home destinations like nursing homes also increased with the degree of frailty, suggesting increased post-acute dependency and functional decline [28–30].

The rate of reported AEs increased consistently with the degree of frailty. However, a slight decline was noticed in the severely frail patient among multiple AE types, possibly due to patient’s limited mobility and dependency on others for personal care, which might have lowered their risks of some AEs like in-hospital falls, self -harm and hospital injuries [10]. The slightly lower incidence of some AEs in the severely frail group may also partly reflect competing risks such as early in-hospital mortality, which reduces the time at risk for certain AE types. Therefore, this finding should be interpreted cautiously and not necessarily as evidence of lower true risk.

Self-harm, patient abuse and adverse drug reaction were rarely reported. This could be due to underreporting, which is a common issue in patient safety incidents, explained by a fear of blame and accountability, lack of feedback, or reporting system complexity [31].

The results of this study align with previous studies reporting higher AE incidence in older patients, though many did not explicitly consider frailty [32–34]. Alotaibi *et al*. [20] found in a systematic review that frailty is associated with the incidence of infections, delirium, in-hospital falls, thromboembolism and pressure injuries. However, many of the reported AEs in the review were not captured in the dataset used in this study, likely because AEs are defined and recorded differently in Datix. The issue of AE definition has been noted in previous research [33], and there is a need for a universal reporting standard for measuring outcomes including AEs.

The link between frailty and AEs is likely driven by decreased physiological resilience and greater clinical complexity [24]. Frail patients often face multiple compounding risks such as polypharmacy, cognitive decline, poor nutrition and immobility, which further increase susceptibility to hospital-acquired AEs [35–39]. Hospital acquired functional decline in older people represents a complex interplay between patient and care factors [40, 41]. Our findings are in line with previous research based on a large cohort of approximately 68 000 CFS assessments, which showed that frailty recording varied according to patients’ clinical and demographic characteristics [42].

### Strengths and limitations

This study has several strengths. It used a large dataset of routinely collected hospital admissions, enhancing the clinical relevance of the findings. Frailty was assessed using the CFS, a validated and widely adopted tool in acute care settings [43], improving the interpretability of results. By including a broad range of AE types, the study offers a comprehensive view of how frailty affects patient safety during hospitalisation.

As a retrospective observational study, causality cannot be established and residual confounding may persist. Although the models were adjusted for key demographic and hospital-related factors, data on important clinical variables, such as comorbidity burden, pre-existing dementia, and polypharmacy, were not available in the dataset. ED wait time may represent both a marker of healthcare exposure and a potential mediator between frailty and AEs. Therefore, adjustment for this variable may affect the observed associations. Low event counts in some AE categories (e.g. self-harm, abuse, adverse drug reactions) limited statistical power. Frailty was measured only once at ED presentation, which may not accurately reflect changes that occurred during the hospital stay including worsening frailty as an outcome. Additionally, the single-centre design may limit the generalisability of the findings, as AE incidence and reporting may be influenced by local clinical pathways, staffing models, safety protocols and institutional reporting culture. Furthermore, changes in reporting practices or hospital systems during the study period may have influenced AE reporting independently of patient frailty. These factors may represent sources of residual confounding that should be considered when interpreting the findings. Incident reporting systems are known to have limitations, including underreporting, variation in reporting practices between clinicians, and potential misuse [44]. In addition, reporting thresholds may vary according to patient characteristics, and some events in severely frail patients may be underreported if perceived as expected complications. However, because frailty assessment was more frequently recorded in the older and more clinically complex patients, some selection bias cannot be completely excluded.

## Conclusion

Frailty, measured in the ED using the CFS, is a strong and independent predictor of risk for a wide range of AE types, including in-hospital falls, pressure ulcers, hospital-acquired infections, moisture-associated skin damage, in-hospital injuries, medication errors and discharge or transfer-related incidents. These findings support the importance of early frailty assessment in the ED to help identify older patients at increased risk of hospital-related AEs. Previous research suggests that frailty-informed care pathways, including comprehensive geriatric assessment and multidisciplinary interventions, may help reduce adverse outcomes in older population [45]. Also, recognising frailty may support shared decision-making and discussions with patients and families regarding risks and expected outcomes of hospital admission.

## Supplementary Material

aa-26-0090-File002_afag175
