# Supplementary material for: The association between the degree of frailty and the risk of hospital related adverse events in older acutely admitted patients
Source: Age Ageing. 2026 Jun 18;55(6):afag175. doi: 10.1093/ageing/afag175 (PMC13278776; doi:10.1093/ageing/afag175)
Supplement: aa-26-0090-File002_afag175 [file aa-26-0090-file002_afag175.docx]

# The Association Between the Degree of Frailty and the Risk of Hospital Related Adverse Events in Older Acutely Admitted Patients

## Supplementary Data

Appendix 1 Grouping of AEs by types and its associated Datix descriptors.

| AE Types | Included event as reported in DATIX |
| --- | --- |
| Medication Errors | - Medicine not administered  - Dose or strength was wrong or unclear  - Dispensing of medication issue  - Wrong drug / medicine  - Wrong quantity  - Mismatch between patient and medicine  - Formulation of medication was wrong  - Contra-indication to the use of the medication  - Medication prescribed to which p. had a known allergy  - Expiry date wrong, omitted or passed  - Wrong route for administration of medication  - 10 Times Medication Error  - Wrong / transposed / omitted medicine label  - Omitted medicine or ingredient  - Controlled drug balance discrepancy |
| Adverse Drug Reaction | - Adverse reaction when drug used as intended |
| Falls | - Fall on level ground  - Fall from a height, bed or chair  - Suspected fall  - Fall resulting in head injury  - Fall resulting in fracture |
| Moisture Associated Skin Damage | - Moisture Associated Skin Damage (MASD) |
| Pressure Ulcers | - Hospital Acquired Pressure Ulcer (Awaiting validation)  - Hospital acquired pressure ulcer Validated - No lapses in Care  - Hospital acquired pressure ulcer Validated - Lapses in Care |
| Infections | - Hospital acquired infection  - Wound or Surgical Site Infection  - Influenza (Suspected or Confirmed)  - Clostridium Difficile Toxins (C. Diff) related death  - Norovirus (Suspected or Confirmed)  - Methicillin Resistant Staphylococcus Aureus (MRSA) Bacteraemia  - Invasive Device related infection  - Hazardous and avoidable exposure to infection |
| Patient Abuse | - Physical abuse, assault or violence by staff  - Sexual Abuse by Staff  - Verbal abuse or disruption by staff  - Assault with a weapon by another patient  - Physical abuse, assault or violence by another patient  - Verbal abuse or disruption by another patient |
| Self-Harm | - Self harm  - Attempted suicide, whether proven or suspected |
| Discharge Transfer | - Inappropriate transfer of patient with Dementia/suspected Dementia  - Absconder / missing patient  - Discharge - inappropriate  - Extended stay / episode of care  - Patient discharged with complications  - Unexpected re-admission or re-attendance  - Unplanned admission / transfer to specialist care unit  - Unplanned return to theatre |
| Hospital Injuries | - Trauma related injury (Not Pressure Ulcer)  - Fainting episode/other Medical Cause  - Bed Rails related injury  - Unintended injury in the course of an operation or clinical task  - Injury caused by medical device  - Collision with an object (Not a fall)  - Collision with an object  - Inappropriate patient handling / positioning  - Infusion injury (extravasation)  - Tripped over an object  - Deep Tissue Injury - No lapses in Care  - Deep Tissue Injury - Lapses in Care |

Appendix 2 Classification of discharge destinations into clinical groups

| Clinical discharge category | Original discharge destinations |
| --- | --- |
| Home | - HOME  - HOME OXY VIRTUAL WD  - HOME WITH ICS |
| Hospital transfer / rehabilitation | - NHS HOSP – GENERAL  - NHS HOSP – MATERNITY  - NHS HOSP LEARN DISAB  - PRIVATE HOSPITAL |
| Care home / residential placement | - LOCAL AUTH CARE/RES  - NHS RUN -CARE HOME  - PRIVATE RUN CARE HOM  - TEMPORARY RESIDENCE |
| Hospice | -PRIVATE HOSPICE |
| Secure psychiatric or custodial placement | - NHS HIGH SECURE PSYC  - NHS MEDIUM SECURE  - PRIVATE MEDIUM SECUR  - REPAT FROM HS PSYCH  - PENAL ESTABLISHMENT |
